# Awareness and Knowledge of Glaucoma among Health Workers in Butajira General Hospital, South Ethiopia

**DOI:** 10.4314/ejhs.v32i5.7

**Published:** 2022-09

**Authors:** Asteway Negussie, Abiye M Alemu

**Affiliations:** 1 Department of Ophthalmology, School of Medicine, College of Health Sciences, Addis Ababa University

**Keywords:** Awareness, Health care workers, Knowledge, Glaucoma

## Abstract

**Background:**

Raising awareness and knowledge of glaucoma is a key means of enhancing people alertness, increasing regular eye screening practice, and the chance of identifying undetected cases. Health professionals other than ophthalmologists are often the first point of contact when patients seek medical advice. However, little is known about the awareness and knowledge of glaucoma among health care professionals in Ethiopia. The objective of this study was to determine general awareness and knowledge of glaucoma among health workers in Butajira General Hospital.

**Methods:**

Hospital-based cross-sectional study was conducted at Butajira Hospital in September and October 2021 among health care providers. All health workers except those on leave and the staff in the department of Ophthalmology were included in the study. The awareness and knowledge were assessed using a self-administered questionnaire. Descriptive statistics was computed to describe the study variable, and logistic regression was conducted to see associations between dependent and independent variables. Significant associations were declared at p-values <0.05.

**Result:**

This study showed that 184 (91.1%) had an awareness of glaucoma among the study participants, whereas 8.9% had never heard about glaucoma. Of those who were aware, only 42% have good knowledge. Professionals who had a history of eye examinations and family history of glaucoma were about 3.0 times more likely to have good knowledge.

**Conclusions:**

Fifty-eight percent of clinical professionals from Butajira Hospital had no awareness at all or had poor knowledge about glaucoma. Being a physician, history of eye examinations, and having a family history of glaucoma were factors related to glaucoma knowledge.

## Introduction

Glaucoma is the second cause of avoidable blindness globally and needs long-term care. The asymptomatic nature and the irreversible blindness make glaucoma a public health challenge(1). Worldwide the current number of glaucoma patients is about 80 million (2).

Blindness due to glaucoma is highest in Africa accounting for 15% of global blindness. The situation is worse in Sub-Saharan Africa, where poor awareness and knowledge further compounded the condition (3). In Ethiopia, glaucoma is one of the leading causes of blindness, causing irreversible sight loss. The increasing prevalence of glaucoma is expected to cause a significant economic burden and poor quality of life(2). High glaucoma morbidity among some African communities may be attributed to low trained professionals, low awareness, underutilization of eye care service, and limited treatment facilities (4,5). There are few studies on the magnitude of awareness related to Glaucoma in Ethiopia, and the study done among adult population in Gish-Abay, found 16.8% of study subjects to know well about glaucoma (6).

Raising public awareness and creating knowledge of glaucoma is a crucial means of addressing its devastating consequences by enhancing people's alertness, increasing regular eye screening practice, and increasing the chance of identifying undetected cases(7). Glaucoma is associated with diseases like hypertension and diabetes, and if persons with risk factors are adequately screened for glaucoma, the chance of early detection is presumably increased (8,9).

Doctors and nurses in departments other than ophthalmology are often the first point of entry when patients seek medical advice. Therefore these health care providers must be well-informed about glaucoma, a “silent thief of sight”. Early diagnosis and institution of treatment can reduce visual impairment and blindness since the significant predictor of eventual blindness is a late presentation (4, 5). Despite its significance, there is no study done on awareness and knowledge about glaucoma in health care workers in Ethiopia. This study aimed to address this issue among health workers in Butajira Hospital.

## Methods

A hospital-based cross-sectional study was conducted among health workers in September and October 2021 in Butajira General Hospital, South Ethiopia. The hospital has a total of 250 clinical staff members. Health workers on leave, and nurses working in the Ophthalmology unit were excluded from the study. The study used sample size determination for a single population, assuming about 16.8% of clinical health providers would have good knowledge about glaucoma(6), and taking 5% a margin of error, and adding 10% for non-response a total of 235 health workers may be needed. Taking finite population correction, the study needs a minimum of 128 health providers. Since the hospital has more than half of the sample, the study tried to include all eligible study subjects.

All study participants agreed to participate after explaining about purpose of the study. The respondents were given a chance to refuse or stop at any moment. All respondents were first asked whether they had ever heard of glaucoma before being recruited for the study. Consecutive questions about the source of information and knowledge of glaucoma were asked only if they had responded that they heard. The data was collected from a self-administered structured questionnaire adapted from previous studies (10–13). The questionnaire consists of sociodemographic features, questions about awareness, source of information, and knowledge about glaucoma. The principal investigator did the administration of questionnaire and data collection. The overall knowledge was determined based on 15 knowledge-related questions, and each correct response was given one point. Knowledge scores were categorized into two groups, and a respondent was said to know about glaucoma if he/she scored 50% or more (14,15).

Data was entered and cleaned by using SPSS Version 25. Descriptive statistics like frequency, percentage, mean and standard deviations for continuous variables were performed. Binary analysis was done to see the significant relation of the independent variables with the dependent variables. Then independent variables found significant at p values<0.05 analyzed using multivariate logistic regressions to control the effect of confounding. Significant factors were identified based on P-value less than 0.05.

**Operational definition**: Awareness was defined as having heard of glaucoma before being recruited for the study (10–13). Based on 15 knowledge questions, knowledge was determined and graded as good or poor. A score of ≥ 50% (≥ 7.5/15) was recorded as good knowledge. Otherwise, it was recorded as having poor knowledge (14, 15).

**Ethical clearance**: Ethical clearance for the study was obtained from the department of Ophthalmology, College of Health Sciences, Addis Ababa University.

## Results

Among eligible 210 clinical staff in Butajira General Hospital,202 health workers completed the questionnaire yielding a response rate of 96.2%. The majority of the participants, 139(68.8 %), were 21–30 with the mean age of 29.8 years ± 5.95 SD. Almost half (49.5%) were females, and the remaining 50.5% were males (Table 1).

**Table 1:** Socio-demographic characteristics of health professionals at Butajira General Hospital, South Ethiopia in 2021 (N=202)

Variables	Frequency (%)
**Age group (Mean±SD=29.82±5.95)**	
21–30	139 (68.8)
31–40	52 (25.7)
≥41	11 (5.4)
**Sex**	
Male	100 (49.5)
Female	102 (50.5)
**Level of Education**	
Diploma	34 (16.8)
BSc	143 (70.8)
MD	13 (6.4)
Medical Specialist	12 (5.9)
**Occupation**	
Physician	24 (11.9)
Nurse	110 (54.5)
Midwife	28 (13.9)
Pharmacist	14 (6.9)
Laboratory technician/technologist	14 (6.9)
Other*	12 (6.0)
**Experience in year** **(Mean±SD =5.62±5.2)**	
<=5	121 (59.9)
6–10	57 (28.2)
>10	24 (11.9)

* Anesthetist, Health Officer, Environmental Health Professional, Integrated Emergency Surgery and Obstetrics, Radiographer

The majority (55.6 %) were females, first-degree graduates (77.8%), nurses (55.6%), and had 5 years or less work experience (83.3%). Almost all respondents (94.4%) who replied that they were not aware of glaucoma were 21–30 years (Table 2).

**Table 2:** Socio- demographic characteristics of respondents who were not aware of glaucoma at Butajira General Hospital, South Ethiopia in 2021 (N=18)

Variables	Frequency (%)
**Age group**	
**(Mean±SD=29.82±5.95)**	
21–30	17 (94.4)
31–40	1 (5.6)
**Sex**	
Male	8 (44.5)
Female	10 (55.6)
**Level of education**	
Diploma	4 (22.2)
Nurse	10 (55.6)
Midwife	6 (33.3)
Pharmacist	1 (5.6)
Laboratory technician/technologist	1 (5.6)
**Experience in year** **(Mean±SD =5.62±5.2)**	
<=5	15 (83.3)
6–10	3 (16.7)

**Figure 1:**
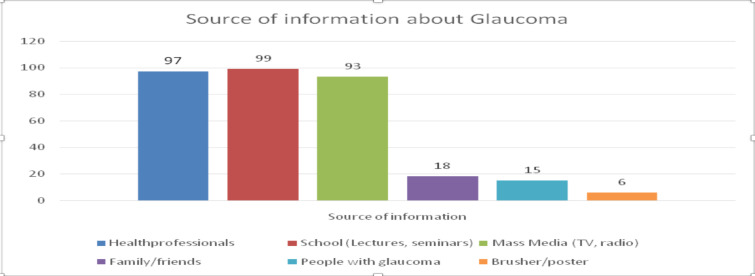
Source of information about Glaucoma at Butajira General Hospital, South Ethiopia in 2021(N=184).

**Figure 2:**
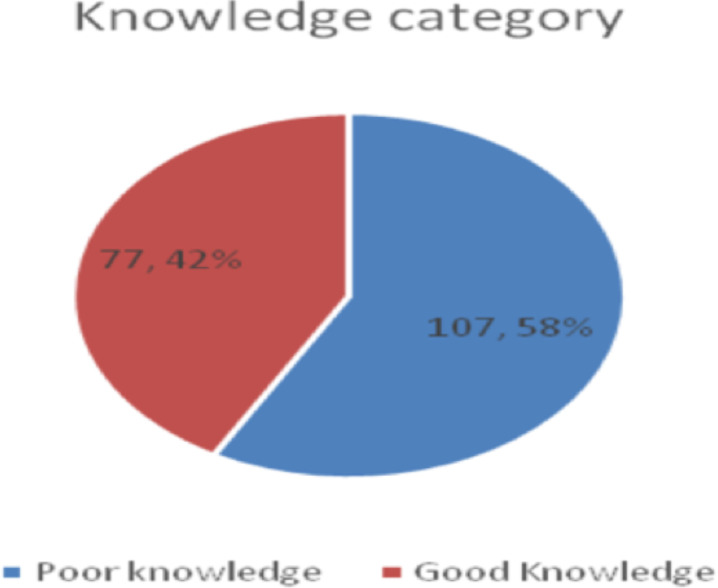
Knowledge of glaucoma among Butajira Hospital health workers, South Ethiopia in 2021 (N=184).

One hundred eighty-four respondents were aware of glaucoma. Their source of information was from school (53.8%) and from other health professionals 97 (52.7%). At the same time, Brushers/ posters were mentioned by only six (Figure 1). One hundred seven (58.2%) responders had poor knowledge (Figure 2).

The majority (78.7%) of participants defined glaucoma as high pressure in the eye. Nineteen (10.3%) did not know about glaucoma. Old age and family history were mentioned by 70.3% and 45.5% as the possible risk factors for glaucoma respectively. The most frequently mentioned symptoms of glaucoma were vision loss (67.3%) followed by tunnel vision (36.1%), while 21 were unable to mention a single symptom.

Eighty-seven (47.3%) of responders think that glaucoma damage is reversible. One hundred twenty participants mentioned medications as a treatment option followed by surgery, which was reported by 116(57.4%). Forty-six (22.8%) mentioned laser as a treatment option. Moreover, almost half (50.5%) of them had an eye examination, and the majority (76.6%) do not have a family history of glaucoma.

Physicians, history of eye examination, and having a family history of glaucoma have shown significant association with glaucoma knowledge. Compared with physicians, nurses and pharmacists were about 20% less likely to have good knowledge. Similarly, other professionals (Laboratory technician/technologist, Anesthetist, Health Officer, Environmental Health Professional, Integrated Emergency Surgery and Obstetrics, Radiographer) were about 10% less likely to have good glaucoma knowledge.

Moreover, professionals who had a history of eye examinations were about 3.4 times more likely to have good glaucoma-related knowledge than their counterparts. Professionals with a family history of glaucoma were about 3.0 times more likely to have good knowledge than those who did not.

## Discussion

In this study, 184(91.1%) respondents were aware of glaucoma. This awareness was lower than the findings of a hospital-based, cross-sectional study among Hospital Personnel in North India (8), Ghana's health science university students (14), and among Nigerian Tertiary Health Care Institution (16), in which all subjects were aware of glaucoma. This difference could be due to differences in the study population as in North India, only nurses and physicians were included while all health professionals were included in this study. Another possible explanation could be related to the content of the curriculum during their training.

The role of health professionals as a source of information was minimal as per the report from a study in Ethiopia's Woliso town, 81.1% got the information from media, but the contribution of health professionals as a source of information was only 5.5% (11). These findings signal the need to integrate glaucoma-related content in health-related disciplines training, which will have double importance to make professionals aware and let other people know.

In this study, school/lectures/ seminars (53.8%), health professionals (52.7%), and media played a central role in creating awareness about glaucoma, while Brushers/ posters were the most negligible 3.3%. A study done in Nigeria among health workers revealed significant sources of information about glaucoma:seminars/lectures 43% and health care personnel twenty percent(17). In Ghana, over half (56.8 %) had acquired their knowledge of glaucoma during their training, and media also played asignifican trole in glaucoma awareness (14).

In this study, the majority (78.7%) defined glaucoma as high pressure in the eye, while about 19(9.4%) didn't know glaucoma's disease entity. Similar findings were reported in a study among Hospital Personnel in North India in which most physicians (80.76%) and nurses (65.26%) understood that glaucoma was associated with high pressure and had an effect on the optic nerve (8). A cross-sectional study done in a Nigerian Tertiary Health Care Institution found that 88.3% of participants knew glaucoma as an increase in eye pressure that damages the back of the eye(16). Another study in Ghana found the majority (86.8%) knew that glaucoma is associated with raised intraocular pressure (IOP) (14).

Contrary to a study among health workers in Nigeria in which 31.7% of participants knew that a family history of glaucoma was a decisive risk factor for developing glaucoma, and 26.7% did not know any risk factors (16). A higher proportion of respondents in this study mentioned risk factors; for instance, old age and family history were mentioned by 70.3% and 45.5%, respectively, while only 5% didn't know any possible risk factors for glaucoma. Similar findings were also reported in North India, in which 76% of physicians and nurses knew that family history was a risk factor(8).

The most frequently mentioned symptoms of glaucoma in this study were vision loss/blindness followed by tunnel vision\ Visual field defect as mentioned by 67.3% and 36.1% of respondents, respectively, while 21(10.4%) were unable to mention a single symptom. This finding was similar to a study in Nigeria among health workers in which 2/3 of the participants mentioned poor vision as the main symptom of glaucoma (17). Surprisingly, the proportion of health workers who mentioned the symptomless nature of glaucoma in this study was low compared to a study done in St.Paul'sHospital in which 78.6% of patients knew that glaucoma is often asymptomatic in its early stages(10)).

Eighty-seven (48%) of respondents in this study reported that they think glaucoma damage is reversible, and this was lower than the studies among health workers in India (61.5%) (8), Nigeria (75.4%) (17) who believed that glaucoma causes irreversible blindness. About 120 (59.4%) of respondents in this study mentioned medications as a treatment option for glaucoma followed by surgery 116(57.4%) and laser 46(22.8%) but only 6 (2.2%) of the study in Nigeria among health workers, knew that laser is used to treat glaucoma (17)

In this study, occupation, history of eye examination, and having a family history of glaucoma were the factors that significantly affect glaucoma-related knowledge. This finding was similar to a study in an urban population of Chennai in south India (13). Age and sex had no significant association with glaucoma knowledge, and similar results were observed in North India (8) and Ghana (14) studies.

The limitation of this study was a single-center, and hence the results cannot be generalized to all health institutions. Even though Butajira Hospital has a department of Ophthalmology, most of the health professionals in this study had no awareness or had poor knowledge about glaucoma. Moreover, physicians, those with a family history of glaucoma and eye examination, were found to have good knowledge. To improve glaucoma knowledge and prevent glaucoma-related irreversible blindness in Ethiopia, health institutions are advised to design and provide continuing medical education on glaucoma. Training centers for health professionals should include glaucoma in their curriculum for long-term solution.
